# Increasing the Safety Profile of the Master Donor Live Attenuated Influenza Vaccine

**DOI:** 10.3390/pathogens9020086

**Published:** 2020-01-29

**Authors:** Thomas A. Hilimire, Aitor Nogales, Kevin Chiem, Javier Ortego, Luis Martinez-Sobrido

**Affiliations:** 1 Department of Microbiology and Immunology, University of Rochester Medical Center, 601 Elmwood Avenue, Rochester, NY 14642, USA; Thomas_hilimire@urmc.rochester.edu (T.A.H.); nogales.aitor@inia.es (A.N.); Kevin_chiem@urmc.rochester.edu (K.C.); 2 Center for Animal Health Research, INIA-CISA, 28130 Valdeolmos, Madrid, Spain; ortego@inia.es

**Keywords:** live attenuated influenza vaccine, influenza, master donor virus, virus–host interaction, vaccine safety, immunogenicity, protection efficacy

## Abstract

Seasonal influenza epidemics remain one of the largest public health burdens nowadays. The best and most effective strategy to date in preventing influenza infection is a worldwide vaccination campaign. Currently, two vaccines are available to the public for the treatment of influenza infection, the chemically Inactivated Influenza Vaccine (IIV) and the Live Attenuated Influenza Vaccine (LAIV). However, the LAIV is not recommended for parts of the population, such as children under the age of two, immunocompromised individuals, the elderly, and pregnant adults. In order to improve the safety of the LAIV and make it available to more of the population, we sought to further attenuate the LAIV. In this study, we demonstrate that the influenza A virus (IAV) master donor virus (MDV) A/Ann Arbor/6/60 H2N2 LAIV can inhibit host gene expression using both the PA-X and NS1 proteins. Furthermore, we show that by removing PA-X, we can limit the replication of the MDV LAIV in a mouse model, while maintaining full protective efficacy. This work demonstrates a broadly applicable strategy of tuning the amount of host antiviral responses induced by the IAV MDV for the development of newer and safer LAIVs. Moreover, our results also demonstrate, for the first time, the feasibility of genetically manipulating the backbone of the IAV MDV to improve the efficacy of the current IAV LAIV.

## 1. Introduction

The influenza A virus (IAV) belongs to the Orthomyxoviridae family of enveloped viruses, which contain an eight-segmented, negative-sense, single-stranded RNA genome [1]. According to the antigenic properties of the hemagglutinin (HA; H1–H18) and neuraminidase (NA; N1–N11) viral surface glycoproteins, IAVs are classified in subtypes with 18 HA and 11 NA subtypes currently circulating [2]. In humans, IAV is responsible for seasonal epidemics that cause mild to severe respiratory illness or even death, representing a threat worldwide to public health. Vaccination is considered the most effective approach to protect against seasonal influenza infections. However, despite worldwide vaccination plans, seasonal influenza is still responsible for 1 billion infections, causing 3–5 million cases of severe disease, and between 290,000 and 650,000 deaths annually, according to the World Health Organization (WHO) and the Center for Disease Control (CDC) [3]. Currently, seasonal IAVs circulating in humans include the H3N2 and the H1N1 subtypes [4]. However due to constant antigenic drift, vaccines have to be reformulated yearly to ensure that the HA and NA proteins included in the vaccine match those present in the seasonally circulating virus.

Inactivated influenza vaccines (IIVs), which induce limited T cell-mediated immune responses [5], have limited protection efficacy when the seasonal vaccine does not match the circulating strains [6,7]. On the other hand, live attenuated influenza vaccines (LAIVs) usually induce more robust adaptive B- and T-cell immune responses associated with better protection against homologous and heterologous influenza viruses [5,6,7]. Despite the multiple advantages of LAIVs, the current LAIV remains restricted for use in healthy children older than two and non-pregnant adults [8]. Therefore, there is a need to develop safer LAIVs that can be used more broadly in the human population, while maintaining their immunogenic properties. Current LAIVs for the treatment of human IAV infections contain the internal genes (*PB2*, *PB1*, *PA*, *NP*, *M*, and *NS*) of a master donor virus (MDV) and the surface HA and NA segments from the seasonal virus strains (H1N1 and H3N2) [9]. The MDV in use in the United States (US) is the A/Ann Arbor/6/60 H2N2 (A/AA/6/60), which was generated after several passages under suboptimal low temperatures [10]. The MDV A/AA/6/60 LAIV has a temperature sensitive (*ts*), cold adapted (*ca*), and attenuated (*att*) phenotype associated with the ability of the virus to replicate in the upper respiratory track (lower temperature) but not in the lower respiratory tract (higher temperatures) [11]. The *ca*, *ts*, and *att* signature of the MDV A/AA/6/60 LAIV is conferred by five mutations in three internal viral genes: the polymerase basic 2 (PB2; N265S) and 1 (PB1; K391E, D581G, and A661T) proteins and the viral nucleoprotein (NP; D34G) [10,12].

IAV has developed several mechanisms to counteract host antiviral responses, namely inhibiting the production of interferon (IFN) and the downstream activities of IFN-stimulated gene (ISG) proteins, which normally inhibit virus replication and propagation [13,14]. Segment 3 (PA) of IAV encodes two proteins, the first being the polymerase acid (PA) protein that is produced directly from the PA mRNA and has endonuclease activity, as well as being a component, together with PB2 and PB1, of the viral polymerase complex [15]. Segment 3 also encodes a second protein, PA-X, which is translated from a +1 frameshift open reading frame (ORF) located in the PA viral segment. PA-X shares the first N-terminal 191 amino acids with PA, but contains a unique short C-terminal sequence [15,16,17,18]. Importantly, PA-X has been shown to have multiple functions, such as the selective degradation of host RNA polymerase II-transcribed mRNAs, which leads to the selective inhibition of cellular protein synthesis, blocking of antiviral responses, or modulating host inflammation [15,19,20,21,22,23]. Despite PA-X and PA sharing the same N-terminal region, PA-X has a stronger endonucleolytic activity, indicating that the C-terminal domain is responsible for the cellular shutoff [24]. Furthermore, the primary transcript produced from the viral genome segment 8 (NS) of IAV is the non-structural protein 1 (NS1), a multifunctional protein which counteracts the innate immune responses, allowing the virus to replicate in IFN-competent systems [14,25,26,27,28]. Synergistically to PA-X, the NS1 protein of certain IAV strains can inhibit host protein synthesis, controlling the expression of IFN and/or ISGs [14,27,29,30,31]. To accomplish this, NS1 binds to the 30 kDa subunit of the cleavage and polyadenylation specificity factor (CPSF30), inhibiting the recognition by the CPSF complex of polyadenylation signals of mRNAs during transcription, blocking the cleavage of immature mRNAs and the addition of the poly (A) tail; this is because this poly (A) tail is required for nucleus export, stability, and translation of cellular mRNAs. The unprocessed mRNAs accumulate in the nucleus, leading to an inhibition of host gene expression, including IFN or ISGs [26,32,33,34]. The amino acid residues responsible for this NS1 function have been mapped in multiple IAV strains. For instance, A/Puerto Rico/8/34 H1N1 cannot bind CPSF30 due to mutations at positions 103 and 106, but that capacity can be restored by introducing amino acid changes at these residues (L103F and I106M) [30,35].

We have postulated that the ability of IAV NS1 and/or PA-X to inhibit innate immune responses might be modulated to generate more effective and/or safer LAIV approaches. In fact, we have generated LAIV-encoding PA-X and NS1 proteins with different abilities to inhibit host gene expression, using the backbone of an A/California/04/09 pandemic (p)H1N1 LAIV, demonstrating the feasibility of implementing this approach, alone or in combination with other methodologies, for the development of a novel LAIVs [18]. Here, we have evaluated whether the current MDV A/AA/6/60 LAIV used for the preparation of the seasonal human LAIV could be improved either in safety and/or immunogenicity by modulating the ability of NS1 and/or PA-X proteins to block host gene expression. To this end, first we evaluated if the NS1 and PA-X proteins of the MDV A/AA/6/60 LAIV have the ability to inhibit host gene expression. Next, we engineered a set of MDV A/AA/6/60 LAIVs encoding PA-X and NS1 proteins with different abilities to inhibit host gene expression, alone or in combination, and the HA and NA of pH1N1. The replication capability of these viruses was evaluated in vitro and their safety, immunogenicity, and protection efficacy against a homologous pH1N1 challenge were assessed in vivo using a mouse model of IAV infection. Our studies indicate that removing PA-X expression by altering the frameshift site, the MDV A/AA/6/60 LAIV can be further attenuated while still retaining a similar immunogenic and protective profile. The implementation of this approach could help to develop safer LAIVs than the currently commercially available IAV LAIV.

## 2. Results

### 2.1. The MDV A/AA/6/60 LAIV PA-X and NS1 Proteins Inhibit Host Gene Expression

**Figure 1:**
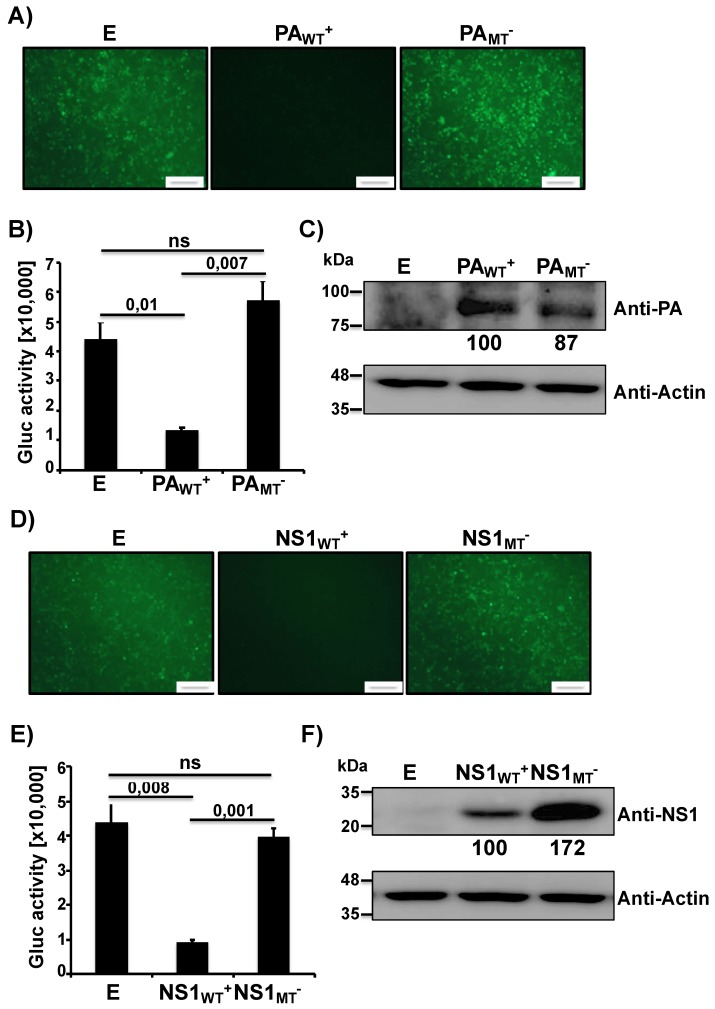
Ability of master donor virus (MDV) A/AA/6/60 live attenuated influenza vaccine (LAIV) PA and NS1 proteins to block host gene expression: Human 293T cells (5 × 10^5^, 12-well plates, triplicates) were transiently co-transfected, using LPF2000, with expression plasmids encoding GFP and Gluc under the control of a chicken beta actin promoter (pCAGGS GFP and pCAGGS Gluc, respectively) together with pDZ plasmids encoding wild-type (WT^+^) or mutant (MT^−^) PA or NS1 proteins; or empty (**E**) plasmid as control. At 24 h post-transfection (p.t.), cells were analyzed by GFP expression (**A**,**D**) under a fluorescent microscope and by Gluc activity (**B**,**E**) from tissue culture supernatants (TCS) using a luminometer. Representative images are shown. Scale bar = 100 μm. Results represent the means and SDs of triplicate values. Protein expression from cell lysates was evaluated by Western blot (**C**,**F**) using specific antibodies for PA (**C**) or NS1 (**F**), or actin as the loading control. Molecular markers are indicated on the left. Western blots were quantified by densitometry using the software ImageJ. Relative band intensities (as described in Materials and Methods) are indicated. ns, not statistical.

Recently, we have shown that IAV PA-X and NS1 proteins can affect viral pathogenicity and the safety and immunogenicity of a pH1N1 LAIV candidate [18]. Therefore, we wanted to examine how PA-X and NS1 of the MDV A/AA/6/60 LAIV affected host gene expression (Figure 1). To this end, we used a well-established approach to evaluate the ability of NS1 and PA-X proteins to block host gene expression [26,27,35,36,37]. Human 293T cells were co-transfected with pCAGGS plasmids expressing the green fluorescent protein (GFP) or Gaussia luciferase (Gluc), along with pDZ plasmids that were either empty (E), contained the WT PA from the MDV A/AA/6/60 LAIV (PA_WT_^+^), or a mutant PA that has an altered frameshift (PA_MT_^−^). At 24 h post-transfection (p.t.), cells were imaged under a fluorescent microscope for GFP expression while tissue culture supernatants (TCS) were collected to quantify Gluc using a luminometer. The PA-X of the MDV A/AA/6/60 LAIV had a strong inhibitory activity showing almost complete knock down of GFP (Figure 1A) and a large decrease in Gluc (Figure 1B) expression. In comparison, the mutant *PA* gene that contained an altered frameshift motif, which is unable to express PA-X, showed no inhibition of GFP or Gluc protein synthesis (Figure 1A,B, respectively). Together these results indicate that the WT PA from the MDV A/AA/6/60 LAIV expresses a functional PA-X that inhibits host gene expression, and that disruption of the frameshift prevents the inhibitory effect mediated by PA-X. To verify that the PA protein was being expressed we also performed Western blot analysis (Figure 1C). We were able to detect similar expression levels of both WT and mutant PA proteins (Figure 1C). We were unable to detect directly PA-X expression in these assays, as the frameshift efficiency is only 1.3% for the WT *PA* gene [15]. Next, we examined the ability of MDV A/AA/6/60 LAIV NS1 to inhibit protein synthesis. Similar to PA-X, we co-transfected the two plasmids expressing the GFP and Gluc reporter genes with either empty (E), WT NS1 (NS1_WT_^+^), or mutant NS1 (NS1_MT_^−^) pDZ plasmids. The WT MDV A/AA/6/60 LAIV NS1 protein was able to effectively inhibit expression of both GFP (Figure 1D) and Gluc (Figure 1E) reporter proteins while the NS1 containing the mutations F103S and M106I in the CPSF30 binding domain showed no inhibitory effect in GFP or Gluc expression (Figure 1D,E, respectively). We also analyzed NS1 protein expression by Western blot (Figure 1F). While both WT and mutant NS1 proteins were expressed from their respective plasmids, the mutant NS1 showed higher expression levels as compared to the WT NS1, because of the ability of WT NS1 to inhibit its own synthesis (Figure 1F) [38]. These results demonstrate that WT NS1 from the MDV A/AA/6/60 LAIV inhibits protein expression and that mutations F103S and M106I, previously described to affect binding to CPSF30 [35], remove its inhibitory effect on host gene expression.

**Figure 2:**
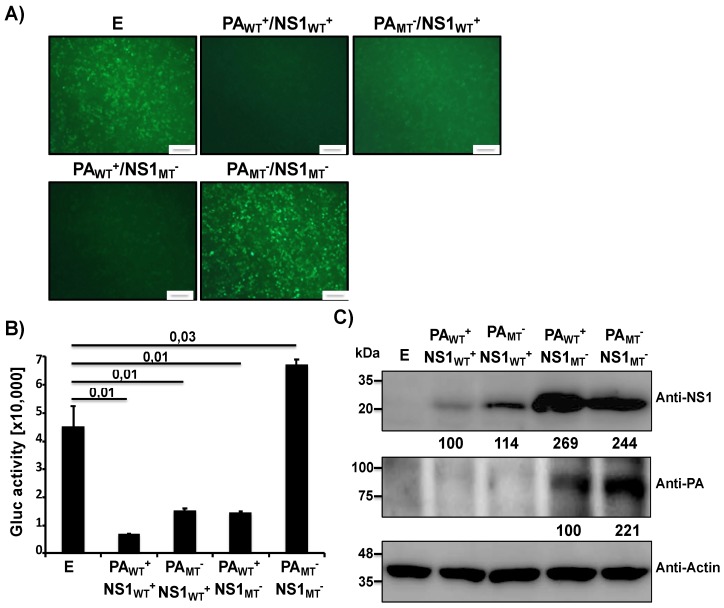
Ability of MDV A/AA/6/60 LAIV PA-X and NS1 proteins to block host gene expression in combination: Human 293T cells (5 × 10^5^, 12-well plates, triplicates) were transiently co-transfected, using LPF2000, with pCAGGS GFP and pCAGGS Gluc plasmids together with the indicated combination of the pDZ plasmids encoding WT^+^ or MT^−^ PA and NS1 proteins; or empty (**E**) plasmid as control. At 24 h p.t., cells were analyzed for GFP expression under a fluorescent microscope (**A**) and for Gluc activity in the TCS using a luminometer (**B**). Representative images are shown. Scale bar = 100 μm. Results represent the means and SDs of triplicate values. Protein expression from cell lysates was evaluated by Western blot using specific antibodies for PA and NS1 proteins (**C**). Actin was used as the loading control. Molecular markers are noted on the left. Western blots were quantified by densitometry using the software ImageJ. Relative band intensities (as described in Materials and Methods) are indicated.

Since both PA-X and NS1 are expressed together during viral infection, we next evaluated the inhibitory effects of PA-X and NS1 from MDV A/AA/6/60 LAIV in combination as well as the inhibitory effect if one or both had mutations that knocked out their function (Figure 2). To that end we co-transfected human 293T cells with the pCAGGS plasmids expressing GFP and Gluc along with a combination of either WT or mutant PA and NS1 pDZ expression plasmids. After 24 h p.t., GFP (Figure 2A) and Gluc (Figure 2B) expression levels were analyzed as described in Figure 1. The combination of both WT PA and NS1 proteins was the most effective at inhibiting host gene expression, while having one functional protein was still effective in significantly lowering reporter gene expression. Removing the functions of both viral proteins led to full expression of the reporter genes. Taken together these results demonstrate that we can tune the amount of inhibition of host cell protein synthesis in the MDV A/AA/6/60 LAIV by altering the ability of PA-X and NS1 proteins to inhibit host gene expression.

### 2.2. Generation of Recombinant WT and Mutant MDV A/AA/60 LAIVs Containing Modified PA and/or NS

**Figure 3:**
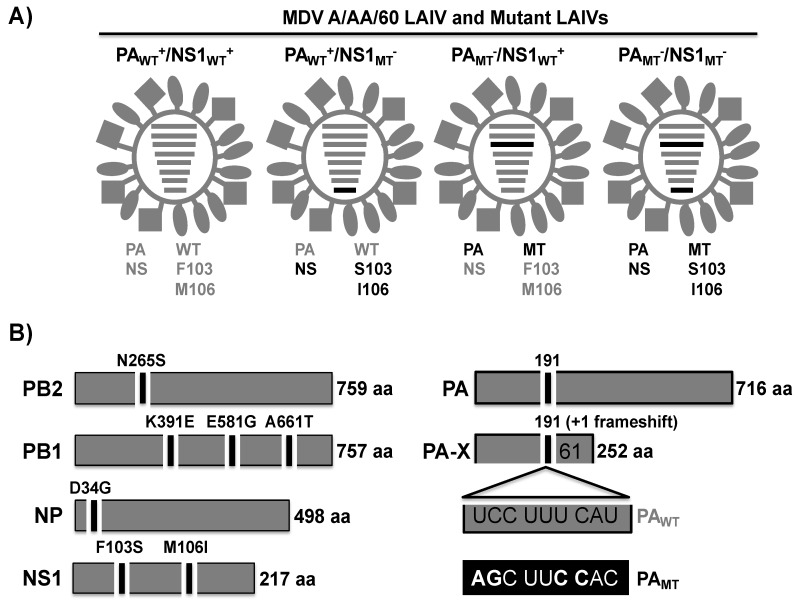
Schematic representation of the recombinant MDV A/AA/6/60 LAIVs. (**A**) Schematic representation of the MDV A/AA/6/60 WT and mutant LAIVs and their gene constellations: Recombinant A/AA/6/60 LAIVs with WT^+^ and MT^−^ segments are indicated with gray or black lines, respectively. PA_WT_^+^/NS1_WT_^+^: virus containing WT PA (UCC UUU CAU) and NS1 (F103/M106) proteins. PA_WT_^+^/NS1_MT_^−^: virus containing WT PA and mutant NS1 (S103/I106) proteins. PA_MT_^−^/NS1_WT_^+^: virus containing mutant PA (AGC UUC CAC) and WT NS1 proteins. PA_MT−_/NS1_MT+_: virus containing both PA and NS1 (mutant proteins. WT^+^ indicates viral proteins (PA and/or NS1) with the ability to inhibit host gene expression. MT^−^ indicates viral proteins (PA or NS1) unable to inhibit host gene expression. (**B**) Schematic representation of PB2, PB1, NP, PA, and NS1 viral proteins: A/AA/6/60 PB2, PB1, NP, and NS1 proteins (left) with the residues mutated to generate the MDV A/AA/6/60 LAIVs indicated. The WT and MT PA or PA-X viral proteins (right) and the mutations introduced into the frameshift motif (PA_MT_) to abolish PA-X expression are shown. Numbers on the right indicate the amino acid (aa) length of the viral proteins.

In order to examine how inhibition of host gene expression affects the safety and immunogenicity of the MDV A/AA/6/60 LAIV, we built a plasmid-based reverse genetic system that we could manipulate to generate the recombinant viruses (Figure 3). To this end we started with the plasmids encoding the six internal proteins from the MDV A/AA/6/60 LAIV, which contain the 5 mutations (PB2 N265S; PB1 K391E, E581G, A661T; and NP D34G) that confer the *ca* and *ts* phenotype to the MDV A/AA/6/60 LAIV (Figure 3A) [10,11]. We then introduced synonymous mutations into the *PA* gene at the frameshift (Figure 3B), resulting in a PA expression plasmid that no longer generates PA-X (PA_MT_^−^) [15,18,19,21,23,39]. Last, we introduced two mutations into the *NS1* gene (F103S and M106I) to change amino acids that have been shown in other IAV strains to be necessary to bind CPSF30 and carry out the host gene expression inhibitory function of NS1 (Figure 3B) [35]. This set of plasmids allows us to look at the inhibitory effects of PA-X and NS1 separately and together during viral infection, allowing us to rescue the WT MDV A/AA/6/60 LAIV (PA_WT_^+^/NS1_WT_^+^), and three mutant MDV A/AA/6/60 LAIVs that have altered PA-X and/or NS1 functions (PA_WT_^+^/NS1_MT_^−^, PA_MT_^−^/NS1_WT_^+^, PA_MT_^−^/NS1_MT_^−^) (Figure 3A).

### 2.3. Growth Kinetics of Recombinant WT and Mutant MDV A/AA/6/60 LAIVs

**Figure 4:**
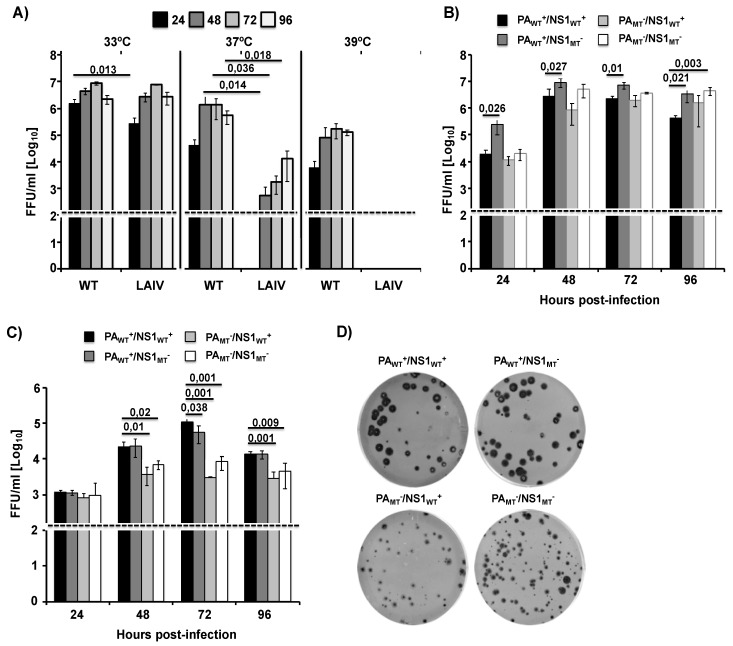
Multicycle growth kinetics and plaque assay of WT and mutant MDV A/AA/6/60 LAIVs. (**A**) Viral growth kinetics of WT A/AA/6/60 and MDV A/AA/6/60 LAIV at different temperatures: TCS from MDCK cells (5 × 10^5^, 12-well plates, triplicates) infected at low multiplicity of infection (MOI, 0.001) with WT A/AA/6/60 or MDV A/AA/6/60 LAIV at 33 °C, 37 °C, and 39 °C were analyzed at the indicated h p.i. (24, 48, 72, and 96) by immunofocus assay using an anti-NP mAb (HB-65). Data represent the means and SDs of the results determined from triplicate wells. The dashed line indicates the limit of detection (200 fluorescent forming units, FFU/mL). (**B**,**C**) Viral growth kinetics of WT MDV A/AA/6/60 LAIV (PA_WT_^+^/NS1_WT_^+^) and mutant MDV A/AA/6/60 LAIVs (PA_WT_^+^/NS1_MT_^−^, PA_MT_^−^/NS1_WT_^+^, and PA_MT_^−^/NS1_MT_^−^): MDCK (**B**) and A549 (**C**) cells (5 × 10^5^, 12-well plates, triplicates) were infected (MOI of 0.001 and 0.025, respectively) with the indicated viruses and incubated at 33 °C. TCS were collected at the indicated h p.i. and viral titers were determined by immunofocus assay. Data represent the means and SDs of the results determined from triplicate wells. Dashed lines indicate the limit of detection (200 FFU/mL). *, *p* < 0.05 using Student’s t test from Microsoft Excel. (**D**) Plaque assay: MDCK cells (1 × 10^6^, 6-well plates) were infected with the indicated MDV A/AA/60 LAIVs and incubated at 33 °C for 3 days. Plaque phenotypes were visualized by immunostaining using the anti-NP mAb HB-65.

Next, we sought to determine how the recombinant WT MDV A/AA/6/60 LAIV and the mutant MDV A/AA/6/60 LAIVs with altered ability to inhibit host gene expression affected viral replication in vitro (Figure 4). To this end, we rescued five recombinant viruses: WT A/AA/6/60, MDV A/AA/6/60 LAIV (PA_WT_^+^/NS1_WT_^+^), and three mutant MDV A/AA/6/60 LAIVs (PA_WT_^+^/NS1_MT_^−^, PA_MT_^−^/NS1_WT_^+^, and PA_MT_^−^/NS1_MT_^−^), all of which express the HA and NA from pH1N1. In MDCK cells infected at low multiplicity of infection (MOI, 0.001), we observed that, compared to WT A/AA/6/60, the MDV A/AA/6/60 LAIV showed no growth at 39 °C and has reduced viral fitness at 37 °C, while maintaining robust growth at 33 °C, indicating that our reverse genetic system successfully rescued a functional *ts, ca* MDV A/AA/6/60 LAIV (Figure 4A). Next we examined the growth kinetics of the MDV A/AA/6/60 LAIV (PA_WT_^+^/NS1_WT_^+^), and the three mutant MDV A/AA/6/60 LAIVs (PA_WT_^+^/NS1_MT_^−^, PA_MT_^−^/NS1_WT_^+^, and PA_MT_^−^/NS1_MT_^−^) in MDCK and human A549 cells at 33 °C. In MDCK cells, all four viruses grow to similar titers but with different peaks of infection (Figure 4B). However, in A549 cells, the viruses lacking PA-X were significantly impaired in viral replication at later time points as compared to the two viruses encoding PA-X (Figure 4C). Moreover, when we examined the plaque morphology of the MDV A/AA/6/60 LAIV (PA_WT_^+^/NS1_WT_^+^) and the three mutant MDV A/AA/6/60 LAIVs (PA_WT_^+^/NS1_MT_^−^, PA_MT_^−^/NS1_WT_^+^, and PA_MT_^−^/NS1_MT_^−^), we saw that the viruses that do not express PA-X (PA_MT_^−^/NS1_WT_^+^ and PA_MT_^−^/NS1_MT_^−^) had plaques that were smaller compared to the two viruses encoding a WT PA-X (Figure 4C). In comparison, viruses which had a mutant NS1 and a functional PA-X (PA_WT_^+^/NS1_MT_^−^) had no change in plaque morphology as compared to the MDV A/AA6/60 LAIV (PA_WT_^+^/NS1_WT_^+^). These data suggest that PA-X is necessary for proper viral replication of the MDV A/AA/6/60 LAIV in vitro in both MDCK and A549 cells.

### 2.4. In Vivo Safety of MDV A/AA/6/60 LAIVs with Varying Ability to Inhibit Host Gene Expression

**Figure 5:**
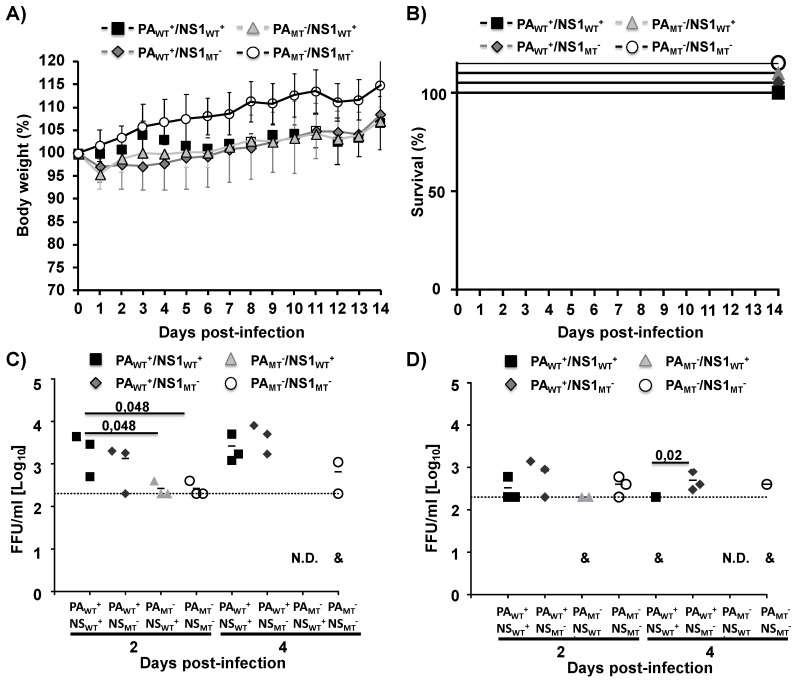
Safety profile of the WT MDV A/AA/6/60 LAIV and mutant MDV A/AA/6/60 LAIVs in vivo: Four-to-six-week-old female C57BL/6 mice (*n* = 11) were infected i.n. with 1 × 10^5^ FFU of the WT MDV A/AA/6/60 LAIV (PA_WT_^+^/NS1_WT_^+^) or the mutant MDV A/AA/6/60 LAIVs (PA_WT_^+^/NS1_MT_^−^, PA_MT_^−^/NS1_WT_^+^, and PA_MT_^−^/NS1_MT_^−^). Body weight (**A**) and survival (**B**) were monitored for 14 days (*n* = 5). At Days 2 and 4 p.i., C57BL/6-infected mice were sacrificed (*n* = 3 per time point) and viral titers were determined in lungs (**C**) and nasal turbinates (**D**) by immunofocus assay using the anti-NP mAb HB-65. Symbols represent data from individual mice. Bars represent the mean for each group and indicates that the virus was only detected in one or two of the infected mice. N.D. indicates that virus was not detected. Dotted lines represent the limit of detection of the assay (200 FFU/mL).

Next, we determined the safety profile of the mutant MDV A/AA/6/60 LAIVs (PA_WT_^+^/NS1_MT_^−^, PA_MT_^−^/NS1_WT_^+^, and PA_MT_^−^/NS1_MT_^−^) as compared to the WT MDV A/AA/6/60 LAIV (PA_WT_^+^/NS1_WT_^+^) in vivo (Figure 5). C57BL/6 mice were infected i.n. with 1 × 10^5^ FFU of the indicated viruses and evaluated morbidity (body weight) (Figure 5A) and mortality (survival) (Figure 5B) for 14 days. We also examined viral titers in the lungs and nasal turbinates of C57BL/6-infected mice at 2 and 4 days p.i. (Figure 5C,D, respectively). C57BL/6 mice are a validated animal model of influenza infection, which has been used previously to evaluate IAV virulence, attenuation, and/or protection efficacy of new influenza vaccines [17,18,26,27,40,41]. None of the viruses caused weight loss, with all groups gaining weight over the course of the experiment (Figure 5A), although the weight variation was not directly compared to mock-infected animals. Moreover, as expected, all infected mice survived viral infection (Figure 5B). Notably, the WT MDV A/AA/6/60 LAIV, containing a mutated PA and a functional NS1 (PA_MT_^−^/NS1_WT_^+^) had undetectable viral titers in both the lungs (Figure 5C) and nasal turbinates (Figure 5D) at Day 4. These data, along with the previous results, suggest that removing PA-X results in a vaccine that is attenuated as compared to the WT MDV A/AA/6/60 LAIV, suggesting that a mutant MDV A/AA/6/60 LAIV lacking PA-X has a better safety profile than the currently MDV A/AA/6/60 LAIV.

### 2.5. Induction of Humoral Immune Responses by WT and Mutant MDV A/AA/6/60 LAIVs

**Figure 6:**
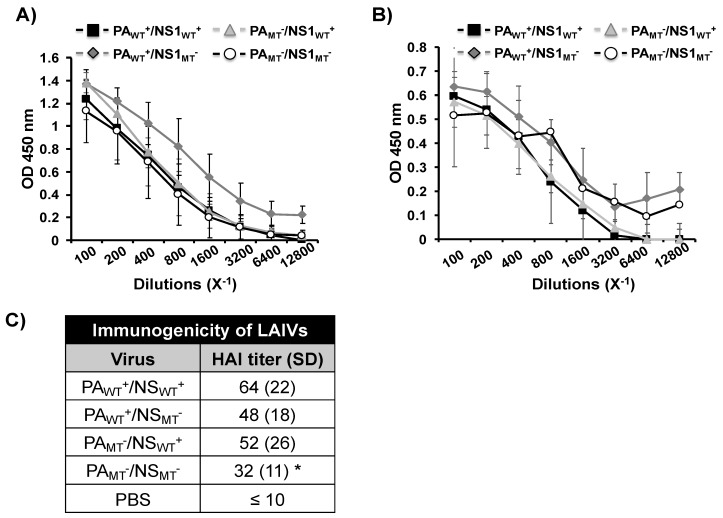
Humoral responses induced by the MDV A/AA/6/60 LAIVs: Four-to-six-week-old female C57BL/6 mice (*n* = 5) were infected i.n. with 1 × 10^5^ FFU with the WT MDV A/AA/6/60 LAIV (PA_WT_^+^/NS1_WT_^+^) or the mutant MDV A/AA/6/60 LAIVs (PA_WT_^+^/NS1_MT_^−^, PA_MT_^−^/NS1_WT_^+^, and PA_MT_^−^/NS1_MT_^−^) as indicated. At 21 days p.i., mice were bled, and sera were collected and evaluated for the presences of antibodies against recombinant pH1N1 HA (**A**) or total viral proteins using cell extracts from pH1N1 virus-infected MDCK cells (**B**) by ELISA. OD, optical density. (**C**) HAI titers were calculated from mouse serum. *, *p* < 0.05 (PA_WT_^+^/NS1_WT_^+^ vs. the other viruses) using a Student’s t test from Microsoft Excel.

Following the safety studies, we next determined if the mutant MDV A/AA/6/60 LAIVs (PA_WT_^+^/NS1_MT_^−^, PA_MT_^−^/NS1_WT_^+^, and PA_MT_^−^/NS1_MT_^−^) induced similar humoral immune responses than that of the WT MDV A/AA/6/60 LAIV (PA_WT_^+^/NS1_WT_^+^) (Figure 6). To this end, we collected blood from mice infected as shown in Figure 5, 21 days after infection and measured, using ELISA, the antibody responses against the viral H1 protein from pH1N1 (Figure 6A), as well as to all viral proteins using cell lysates from pH1N1-infected MDCK cells (Figure 6B). All the mutant MDV A/AA/6/60 LAIVs (PA_WT_^+^/NS1_MT_^−^, PA_MT_^−^/NS1_WT_^+^, and PA_MT_^−^/NS1_MT_^−^) induced an overall antibody response against the viral HA and other viral proteins that was comparable to that induced by the WT MDV A/AA/6/60 LAIV (PA_WT_^+^/NS1_WT_^+^) (Figure 6A,B, respectively). Next, we determined the ability of these antibodies to inhibit HA activity, using an hemagglutination inhibition (HAI) assay (Figure 6C). While all four MDV A/AA/6/60 LAIVs induced HAI antibodies, the MDV A/AA/6/60 LAIV containing both mutant PA and NS1 proteins (PA_MT_^−^/NS1_MT_^−^) induced significantly less HAI antibodies than the WT MDV A/AA/6/60 LAIV (PA_WT_^+^/NS1_WT_^+^).

### 2.6. Protective Efficacy of MDV A/AA/6/60 LAIVs Against a Homologous pH1N1 Viral Challenge

**Figure 7:**
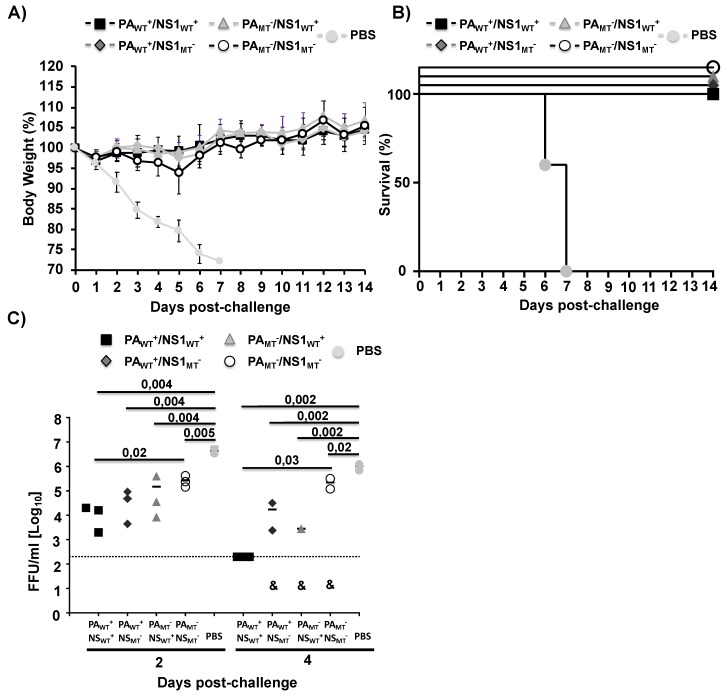
Protective efficacy of the MDV A/AA/6/60 LAIVs: Four-to-six-week-old female C57BL/6 mice (*n* = 11) were infected i.n. with 1 × 10^5^ FFU of the WT MDV A/AA/6/60 LAIV (PA_WT_^+^/NS1_WT_^+^) or mutant MDV A/AA/6/60 LAIVs (PA_WT_^+^/NS1_MT_^−^, PA_MT_^−^/NS1_WT_^+^, PA_MT_^−^/NS1_MT_^−^), or PBS. At 21 days post-vaccination mice were challenged with 1000X MLD_50_ of pH1N1. Body weight (**A**) and mortality (**B**) were monitored for 14 days (*n* = 5). At Days 2 and 4 post-challenge, mice were sacrificed (*n* = 3 per time point) and viral titers in the lungs of infected animals were determined by immunofocus assay using the anti-NP mAb HB-65 (**C**). Symbols represent data from individual mice. Bars represent the geometric mean for each group and the indicated virus was detected only in one or two mice. The dotted line represents the limit of detection (200 FFU/mL).

Finally, we determined the protective efficacy of the three mutant MDV A/AA/6/60 LAIVs (PA_WT_^+^/NS1_MT_^−^, PA_MT_^−^/NS1_WT_^+^, and PA_MT_^−^/NS1_MT_^−^) as compared to the WT MDV A/AA/6/60 LAIV (PA_WT_^+^/NS1_WT_^+^) (Figure 7). To that end, mice were vaccinated with 1 × 10^5^ FFU of the MDV A/AA/6/60 LAIVs and challenged 21 days p.i. with 1000X the mouse lethal dose 50 (MLD_50_) of pH1N1 [42] and monitored for changes in body weight (Figure 7A) and survival (Figure 7B) for 14 days. We also evaluated the presence of the pH1N1 virus challenge in the lungs of infected animals at Days 2 and 4 post-challenge (Figure 7C). None of the vaccinated mice lost weight (Figure 7A) and all survived (Figure 7B) the lethal challenge with pH1N1, contrary to the situation of the mock (PBS)-vaccinated mice that all succumbed to viral infection. Mice that were vaccinated with the MDV A/AA/6/60 LAIV that had both mutant PA and NS1 (PA_MT_^−^/NS1_MT_^−^) showed significantly higher viral titers at Days 2 and 4 post-challenge compared to the mice vaccinated with the WT MDV A/AA/6/60 LAIV (Figure 7C), but significantly less than the PBS-treated control group, indicating that while there was a protective immune response (no mice died) it was not as robust, in agreement with the previous results showing reduced HAI antibodies (Figure 6C). The WT MDV A/AA/6/60 LAIV (PA_WT_^+^/NS1_WT_^+^) and the two mutant MDV A/AA/6/60 LAIVs containing only one mutant protein (PA_WT_^+^/NS1_MT_^−^ and PA_MT_^−^/NS1_WT_^+^) showed a significant reduction in viral titers on both days as compared to the PBS control treated mice (Figure 7C). Taken together these results indicate that altering the ability of the WT MDV A/AA/6/60 LAIV to inhibit host protein synthesis does not abolish protective efficacy while improving its safety profile.

## 3. Discussion

IAV infection requires a fine balance between inhibiting the host cell antiviral mechanisms by shutting down translation, and allowing enough of the host machinery active to allow for viral replication [16]. Since the discovery that PA-X and NS1 can both inhibit host gene expression, it has been seen that the different strains of IAV use each protein differently [16]. For example, pH1N1 uses PA-X exclusively for inhibiting host protein synthesis as its NS1 protein does not possess this function [17,18]. Furthermore, it has been shown that removing PA-X has different effects in different virus backbones. Removing PA-X expression in 1918 H1N1 [15], pH1N1 [39], and H9N2 [20] IAVs reduces viral pathogenicity, while removing PA-X in H5N1 IAV increases viral replication and pathogenicity [21]. These differing effects, together with our recent findings with pH1N1 [17,18] led us to believe that altering PA-X and/or NS1 function in the context of the WT MDV A/AA/6/60 LAIV would be a viable option to tune the safety, immunogenicity, and protection efficacy conferred by the LAIV. However, the possible side effects linked to changes in the ability of a new MDV A/AA/6/60 LAIV to modulate host innate immune responses, such as low immunogenicity, should be evaluated in other animal models of influenza infection. For instance, the implication in virus transmission should be tested in ferrets or guinea pigs, both well-stablished animal models of influenza virus infection and transmission [43,44,45,46].

The current MDV A/AA/6/60 LAIV has mutations mainly located in areas of the polymerase complex. There is always a concern that, with enough pressure, the MDV A/AA/6/60 LAIV could introduce mutations to compensate and revert back to a pathogenic strain [47]. By introducing mutations into additional viral genes (*PA* and *NS1*), the probability of reversion to a more virulent strain is decreased even further, adding to the increased safety profile desired for an MDV LAIV. However, the stability of the generated viruses should be tested using validated systems for IAV vaccine production. Additionally, by having another attenuation marker outside of the current PB2, PB1 and NP viral segments, there is less concern about the MDV A/AA/6/60 LAIV reassorting with a circulating strain.

In this work we demonstrate, for the first time, that the human MDV A/AA/6/60 LAIV has both active PA-X and NS1 proteins that can inhibit host gene expression (Figure 1). Building on our previous work, we also showed that the effects of these two viral proteins is additive in a reporter assay (Figure 2). We also demonstrate the feasibility of generating recombinant MDV A/AA/6/60 LAIVs with altered PA-X and/or NS1 proteins (Figure 3). Removing PA-X, however, showed to be detrimental for viral replication and resulted in a smaller plaque phenotype in cultured cells (Figure 4). Conversely, removing the inhibitory function of NS1 did not adversely affect viral replication of the MDV A/AA/6/60 LAIV (Figure 4). These trends were also seen in vivo. While all the MDV A/AA/6/60 LAIVs were still fully attenuated and did not result in any weight loss in mice, viruses lacking PA-X showed reduced viral replication in lungs and nasal turbinate, while those lacking a functional NS1 resembled the parental WT MDV A/AA/6/60 LAIV (Figure 5). Even with the reduced replication seen in some of these viruses, all of them were able to induce similar protective antibody responses (Figure 6) that provided full protection against a lethal homologous challenge with pH1N1 (Figure 7). However, while all of the MDV A/AA/6/60 LAIVs were protective against weight loss and mortality, there were differences in the amount of pH1N1 detected in the lungs of challenged mice at Days 2 and 4 p.i., with the parental WT MDV A/AA/6/60 LAIV showing the least amount of virus in the lungs and the double mutant MDV A/AA/6/60 LAIV (PA_MT_^−^/NS1_MT_^−^) showing the most pH1N1 viral replication (Figure 7).

Taken together our results show that removal of PA-X by modifying the frameshift sequence results in a vaccine that is fully protective but is further attenuated, suggesting that in a clinical setting this vaccine would be safer than the current MDV A/AA/6/60 LAIV in use. Thus, this new MDV A/AA/6/60 LAIV with reduced PA-X levels could be used for parts of the population where current LAIVs are not recommended, such as children under the age of two, immunocompromised individuals, the elderly, and pregnant adults. Furthermore, one of the major drawbacks for the current LAIV is that it can have adverse effects in children younger than two years of age, a cohort that would benefit greatly from an LAIV over the traditional chemically IIV. In addition, this safer MDV A/AA/6/60 LAIV lacking a functional PA-X will have another attenuation marker outside the current PB2, PB1, and NP segments, leading to less concerns of reversion to a WT virulent phenotype.

This and our previous work on the interplay of NS1 and PA-X in pH1N1 [17,18] show that modifying the functions of the PA-X and NS1 proteins is a viable way to fine tune the attenuation of vaccine candidates. Vaccination is the best way to prevent the spread of influenza and reduce the global health burden. However, in recent years the vaccine efficacy has been reported to be less than 50% leading to a large effort to find alternative vaccines that work better. This work demonstrates one method of increasing the safety profile of the current IAV LAIV without losing any of its protective efficacy that could be implemented with new universal vaccines strategies that are being currently developed for the treatment of influenza viral infections.

## 4. Materials and Methods

### 4.1. Cells and Viruses

Human embryonic kidney 293T (ATCC CRL-11268), human lung epithelial carcinoma A549 (ATCC CCL-185), and Madin-Darby canine kidney, MDCK (ATCC CCL-34) cells were grown and maintained in Dulbecco’s modified Eagle’s medium (DMEM) (Mediatech, Inc.) supplemented with 5% fetal bovine serum (FBS) (Atlanta Biologicals) and 1% penicillin (100 U/mL)–streptomycin (100 μg/mL)–2 mM L-glutamine, P-S-G (Mediatech, Inc.) at 37 °C in air enriched with 5% CO_2_. 

The recombinant wild-type (WT) A/California/4_NYICE_E3/2009 pH1N1 virus has been previously described [43]. The recombinant WT A/Ann Arbor/6/60 H2N2 (A/AA/6/60), MDV A/AA/6/60 LAIV, and mutant MDV A/AA/6/60 LAIVs were generated using plasmid-based reverse genetics as described below.

### 4.2. Plasmids

To generate the recombinant WT A/AA/6/60, MDV A/AA/6/60 LAIV, and mutant MDV A/AA/6/60 LAIVs, recombinant PB2 LAIV, PB1 LAIV, PA, and NP viral segments were synthesized de novo (Geneart) into a pMK plasmid. The recombinant M and NS segments were synthetized using Strings DNA Fragments technology (Geneart) and cloned into a pGEM-T plasmid (Promega). All the viral genes were synthetized with appropriate restriction sites at the 5′ and 3′ ends for subcloning into the ambisense plasmid pDZ [48]. To generate PB2 WT and PB1 WT, the *ts* mutations in PB2 (S265N), PB1 (E391K, G581E, and T661A) and NP (G34D) were introduced [49,50,51]. PA-X-deficient plasmids were created by mutating the frameshift motif from UCC UUU CAU (PA_WT+_) to AGC UUC CAC (PA_MT_^−^) [18]. Two amino acid substitutions (F103S and M106I) [30,35] were introduced into the viral NS segment to mutate the NS1 protein. All the mutations were introduced by site-directed mutagenesis in the pMK or pGEM-T plasmids before subcloning into the ambisense pDZ plasmid. All plasmids were confirmed by sequencing (ACGT Inc.). Primers for the generation of the different plasmid constructs are available upon request.

### 4.3. Viral Rescues

Virus rescues were performed as we described previously [2,40,52]. Briefly, co-cultures (1:1) of 293T and MDCK cells in 6-well plates were co-transfected with 1 μg of each of the ambisense plasmids (pDZ-PB2, −PB1, −PA_WT_^+^ or PA_MT_^−^, −HA, −NP, −NA, −M, and NS_WT_^+^ or NS1_MT_^−^) using Lipofectamine 2000 (LPF2000, Invitrogen). At 12 h post-transfection (p.t.), the transfection medium was replaced with DMEM containing 0.3% bovine serum albumin (BSA), 1% P-S-G, and 0.5 μg/mL of N-tosyl-l-phenylalanine chloromethyl ketone (TPCK)-treated trypsin (Sigma). At 3–4 days p.t., tissue culture supernatants (TCS) were collected, clarified, and used to infect fresh MDCK cells. At 3 to 4 days post-infection (p.i.), recombinant viruses were plaque purified and scaled up in MDCK cells. Stocks were titrated by immunofocus assay (Fluorescence Focus Units (FFU)/mL)) on MDCK cells [2,52]. Virus stocks were confirmed by sequencing the PA and NS1 ORFs using purified total RNA (TRIzol reagent; Invitrogen) from infected MDCK cells according to the manufacturer’s specifications.

### 4.4. Inhibition of Host Gene Expression

To evaluate the effect of MDV A/AA/6/60 LAIV NS1 and PA-X proteins on host protein synthesis, human 293T cells (2.5 × 10^5^ cells/well, 24-well plate format, triplicates) were transiently co-transfected in suspension, using LPF2000, with 1 μg/well (500 ng/plasmid) of the indicated pDZ plasmids encoding WT or mutant PA (PA_WT_^+^ or PA_MT_^−^) proteins, WT or mutant NS1 (NS1_WT_^+^ or NS1_MT_^−^) proteins, together with 25 ng/well of pCAGGS plasmids expressing the green fluorescent protein (GFP) and Gaussia luciferase (Gluc). pDZ empty (E) plasmid was used as control or to complete 1 μg/well when only one NS or PA plasmid was added. At 24 h p.t., cells were analyzed for GFP expression under a fluorescence microscope and Gluc activity was quantified from TCS using a Biolux Gaussia luciferase reagent (New England BioLabs) and a Lumicount luminometer (Packard). The mean value with standard deviations (SDs) were calculated using Microsoft Excel software.

### 4.5. SDS-PAGE and Western Blot Analysis

Transfected cells were lysed in RIPA buffer (25 mM Tris HCl pH 7.6, 150 mM NaCl, 1% NP-40, 1% sodium deoxycholate, 0.1% SDS) and proteins were separated by denaturing electrophoresis using 10% SDS-polyacrylamide gels and transferred to a nitrocellulose membrane. Membranes were blocked for 1 h with 5% dried skim milk in phosphate-buffered saline (PBS) containing 0.1% Tween 20 (T-PBS) and incubated overnight at 4 °C with primary rabbit polyclonal antibodies (pAb) against NS1 [53] or a mix of mouse monoclonal antibodies (mAb) against PA (1F6, NR-19225; 5C5, NR-19226; and 8E10, NR-19224) obtained from BEI Resources. A mouse mAb against actin (A1978; Sigma) was used as an internal loading control. Horseradish peroxidase (HRP) secondary antibodies (GE Healthcare) against either mouse or rabbit immunoglobulins (Ig) were used to detect bound primary antibodies. Protein expression was detected with a SuperSignal West Femto maximum-sensitivity chemiluminescent substrate kit (Thermo Scientific) in accordance with the manufacturer’s instructions. Protein bands were normalized to the level of beta-actin expression, and then the level of expression of the WT protein was considered 100%. In the case of Figure 2C, for the anti-PA analysis, PA_WT_+/NS1_MT_− was considered 100%.

### 4.6. Viral Growth Kinetics

To assess virus growth kinetics in vitro, confluent monolayers of MDCK or A549 cells (4 × 10^5^ cells/well, 12-well plate format, triplicates) were infected at a multiplicity of infection (MOI) of 0.001 (MDCK) or 0.025 (A549). After 1 h of virus adsorption at room temperature, cells were overlaid with DMEM containing 0.3% BSA, 1% P-S-G, and TPCK-treated trypsin (1 μg/mL for MDCK cells and 0.25 μg/mL for A549 cells) and incubated at 33 °C, 37 °C, or 39 °C for WT vs. LAIV and at 33 °C for the PA and NS1 mutants. At the indicated times post-infection, p.i. (24, 48, 72, and 96 h), TCS were collected and viral titers were determined by immunofocus assay (FFU/mL) as previously described [18,40,52]. Briefly, confluent wells of MDCK cells (10^4^ cells/well, 96-well plate format, triplicates) were infected with 10-fold serial dilutions of TCS. At 12 h p.i., cells were fixed and permeabilized (4% formaldehyde and 0.5% Triton X-100 in PBS) for 15 min at room temperature. The cells were then incubated in blocking solution (2.5% BSA in PBS) for 1 h at room temperature and incubated with the influenza virus NP mAb HB-65 (ATCC) [18,40,52] for 1 h at 37 °C. After washing with PBS, cells were incubated with a fluorescein isothiocyanate (FITC)-conjugated rabbit anti-mouse IgG secondary antibody (Dako) for 1 h at 37 °C. NP-expressing positive cells were enumerated to determine the virus titer (FFU/mL). The mean value and SDs were calculated using Microsoft Excel software.

### 4.7. Plaque Assay and Immunostaining

Confluent monolayers of MDCK cells (10^6^ cells/well, 6-well plate format) were infected for 1 h at room temperature, and after virus adsorption, cells were overlaid with agar and incubated at 33 °C. At 3 days p.i., cells were fixed with 4% paraformaldehyde for 15 min at room temperature. After the overlays were removed, cells were permeabilized (0.5% Triton X-100 in PBS) for 15 min at room temperature and prepared for immunostaining as previously described [18,51], using the NP mAb HB-65 and vector kits (Vectastain ABC kit and DAB HRP substrate kit: Vector) following the manufacturer’s specifications.

### 4.8. Mouse Experiments

Female 6-to-8-week-old C57BL/6 mice were purchased from the National Cancer Institute (NCI) and maintained in the animal care facility at the University of Rochester under specific-pathogen-free conditions. All animal protocols were approved by the University of Rochester Committee of Animal Resources and complied with the recommendations in the Guide for the Care and Use of Laboratory Animals of the National Research Council. Mice (*n* = 11/group) were anesthetized intraperitoneally (i.p.) with 2,2,2-tribromoethanol (Avertin; 240 mg/kg of body weight) and then infected intranasally (i.n.) with 30 μL of the indicated LAIVs. For challenge experiments mice (*n* = 11/group) were anesthetized i.p. and infected i.n. with 1000X the Median Lethal Dose (MLD_50_) of pH1N1. Mice (*n* = 5/group) were monitored daily for morbidity (body weight loss) and mortality (survival) as previously described [17,26,27,36,40,41]. Mice showing 25% loss of their initial body weight were considered to have reached the experimental endpoint and were humanely euthanized. Virus replication was evaluated by determining viral titers in the lungs or nasal turbinates at 2 and 4 days p.i. To that end, three mice in each group were sacrificed, and lungs or nasal turbinates were extracted and homogenized. Virus titers were determined by immunofocus assay (FFU/mL) as indicated above.

### 4.9. Enzyme-Linked Immunosorbent Assay (ELISA)

Mouse sera were collected by submandibular bleeding at 21 days p.i. and evaluated for the presence of influenza virus-specific antibodies by ELISA [18,40]. Briefly, 96-well plates were coated for 16 h at 4 °C with lysates from mock- or pH1N1 WT virus-infected MDCK cells. Alternatively, plates were coated with pH1N1 HA (200 ng/well) (FR-180, International Reagent Resource) recombinant protein. After washing with PBS, coated wells were blocked with PBS containing 1% BSA and then incubated with 1:2 dilutions (starting dilution of 1:100) of mouse serum at 37 °C. After 1 h incubation, wells were washed with PBS and incubated with HRP-conjugated goat anti-mouse IgG (GE Healthcare) for 30 min at 37 °C. The reactions were developed with tetramethylbenzidine (TMB) substrate (BioLegend) for 10 min at room temperature, quenched with 2 N H_2_SO_4_, and read at 450 nm (Vmax kinetic microplate reader; Molecular Devices).

### 4.10. Hemagglutination inhibition (HAI) Assay

HAI assays were used to assess the presence of HA inhibition antibodies [18]. To that end, mouse sera were treated with receptor-destroying enzyme (RDE) (Denka Seiken) and heat inactivated for 30 min at 56 °C. Sera were then serially 2-fold diluted (starting dilution of 1:20) in 96-well V-bottom plates and mixed 1:1 with 4 hemagglutinating units (HAU) of pH1N1 WT for 60 min at room temperature. The HAI titers were determined by adding 0.5% turkey red blood cells (RBCs) to the virus-antibody mixtures for 30 min on ice.
